# Non-target embolic events during prostatic embolization with ethylene vinyl alcohol copolymer (EVOH)

**DOI:** 10.1186/s42155-023-00402-w

**Published:** 2023-11-03

**Authors:** Jacques Sédat, Paolo Arnoffi, Florent Poirier, Modhar Jamjoom, Charles Raffaelli, Frederic Colomb, Yves Chau

**Affiliations:** 1 Service de NeuroInterventionnel, CHU de Nice Hôpital, Pasteur 2, 30 Voie Romaine, Nice, France; 2 Hôpital de Grasse. Chemin de Clavary, Service d’urologie, 06130 Grasse, France; 3 Service de NeuroInterventionnel, CHU de Nice, Hôpital, Pasteur 2, 30 Voie Romaine, Nice, France

**Keywords:** Lower urinary tract symptoms, Benign prostate hyperplasia, Prostate artery embolization, Ethylene vinyl alcohol copolymer

## Abstract

**Background:**

This study evaluated nontarget embolization (NTE) during prostatic artery embolization (PAE) with ethylene vinyl alcohol copolymer (EVOH).

**Results:**

Ten consecutive patients treated by PAE with EVOH for the presence of disabling benign prostatic hyperplasia (BPH)-related lower urinary tract symptoms (LUTS) between June 22 and January 2023 were included in this prospective study. The inclusion criteria were as follows: LUTS attributed to BPH, LUTS duration ≥ 6 months, failure to respond to standard pharmacotherapy, IPSS > 18 or QoL score > 2, and prostate volume > 40 mL. Embolization was performed under general anaesthesia. According to established techniques, a microcatheter was positioned bilaterally within the feeding arteries, and EVOH was injected slowly under X-ray control. Unenhanced pelvic computed tomography scans were carried out before and after embolization to assess the NTE. The safety of the prostatic embolization procedure with EVOH was assessed by collecting adverse effects over 3 months of evaluation that included the International Prostate Symptom Score (IPSS) and quality of life (QoL) score.-up evaluations, occurring at 3, 6, and 12months, included International Prostate Symptom Score.

Bilateral PAE was technically successful in 9 patients, and unilateral injection was performed in one patient. The postoperative scanner showed a distribution of the embolization material in the two lobes of the prostate in all patients. The procedure time varied from 120 to 150 (mean: 132) minutes. Eight out of 10 patients developed pollakiuria within 24 h; none of the patients had postoperative pain. Two patients required catheterization for postoperative urinary retention. Catheters were removed successfully at the end of the first day for one of these patients and on the tenth day for the other. At the 3-month follow-up, patients showed significant improvement in the International Prostate Symptom Score (*n* = 10; mean = -11,5; *P* < 0.01) and quality of life score (*n* = 10; mean = -3,40; *P* < 0.01). Only one patient presented one asymptomatic muscular NTE.

**Conclusions:**

PAE with EVOH is safe, effective, and associated with few NTEs and no postoperative pain. Prospective comparative studies with longer follow-ups are warranted.

**Trial registration:**

IDRCB, 2021-AO29-56–35. Registered 27 May 2022, http://clinicaltrials.gov/study/NCT05395299?cond=embolization&term&rank=1.

## Background

Prostatic artery embolization (PAE) with microparticles has been shown to be safe and effective for treating lower urinary tract symptoms (LUTS) in patients with benign prostatic hyperplasia (BPH). If PAE seems to present fewer complications than transurethral resection of the prostate [1, 2], nontarget embolization (NTE) is one of the drawbacks of the technique. According to Brown et al., prostatic particle embolization is associated in all cases with NTE; NTE is responsible for secondary events such as penile ulceration, rectal or anal pain, and postoperative haematuria [3]. Serious complications after prostatic embolization are rare but also mainly have an ischaemic origin [4]. NTE can be explained by vascular anatomical variations, high-flow vascular anastomoses, suboptimal catheter placement and the absence of radiopacity of the particles. Ethylene vinyl alcohol copolymer (EVOH) is a radiopaque and viscous liquid embolic agent that has been used for the endovascular treatment of cerebral arteriovenous malformations since 2005 [5]. Its injection can be halted if extension of the agent into a nontargeted vessel is detected and then resumed once the product has solidified. Prostatic embolization with EVOH may reduce the risk of extraprostatic embolization and ischaemia of a nontarget organ. This study evaluated the safety and the rate of detectable nontarget embolic deposition during PAE with EVOH.

## Methods

**Fig. 1:**
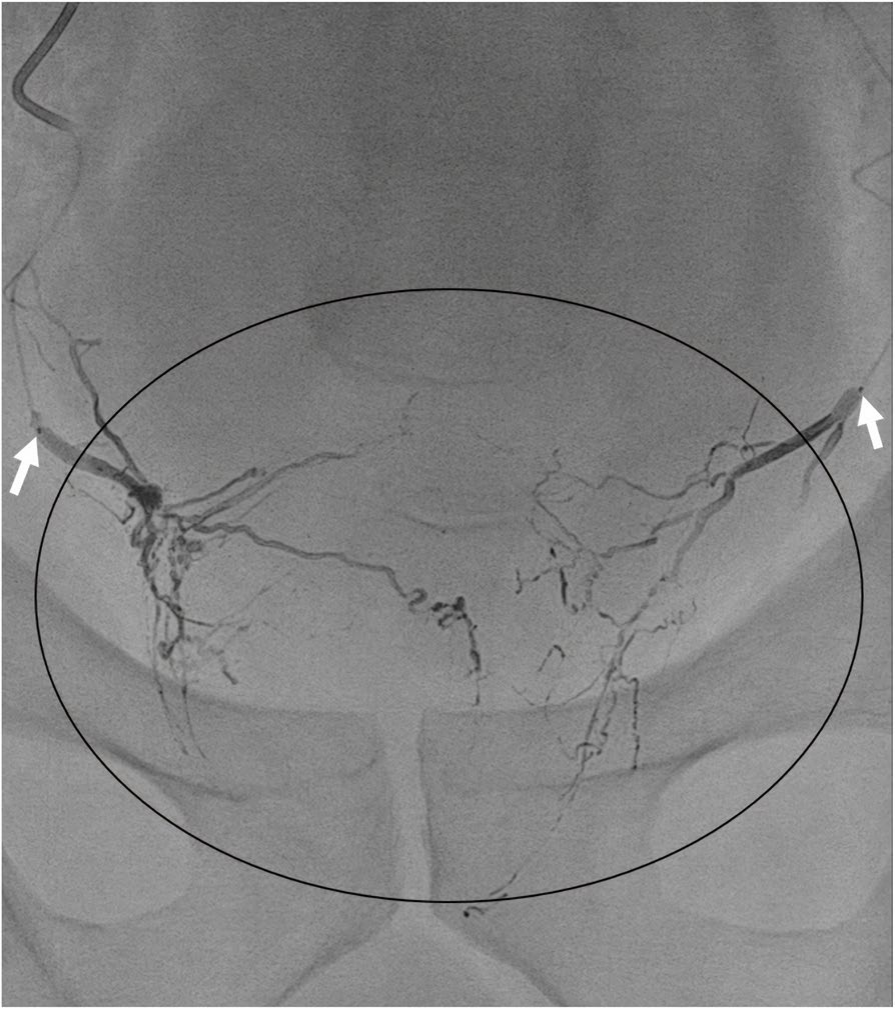
Pelvic AP view after embolization. Legend: in the black circle: bilateral occlusion of the prostatic branches by EVOH. White arrows indicate the tip of the microcatheters

A) Study population
  The study protocol and the consent form were approved by (blinded).
  Ten consecutive patients treated by PAE for disabling BPH-related LUTS were included in this prospective, single-centre, single-arm study (Table 1), performed according to Good Clinical Practice requirements and the Helsinki Declaration, and registered on Clinical Trials.gov (blinded).
B) Endovascular procedure
  All PAE procedures were performed by two experienced interventional radiologists (JS, YC), with 20 and 15 years of experience in vascular embolization, who were familiar with prostate embolization and with the use of endovascular injection of EVOH.
  PAE was performed on an outpatient basis under general anaesthesia with no bladder catheter, usually via a double femoral approach. A 4-French (Fr) sheath (Terumo, Tokyo, Japan) was inserted into the right and left common femoral arteries. Two 4-Fr Berenstein catheters (Cordis Corp, Miami Lakes, Florida) were inserted into both internal iliac arteries after a crossover technique, and prostatic arteries were identified by selective internal iliac arteriography. A 1.3-F Headway Duo microcatheter (MicroVention Europe, Saint Germain en Laye, France) was placed coaxially into each Berenstein catheter and positioned within the feeding arteries.
  The microcatheter tip was placed into the common prostatic artery trunk on both sides. First, the microcatheter dead space was filled with solvent, and then EVOH (SQUID (Balt, Montmorency, France)) was injected under X-ray control slowly and bilaterally according to manufacturer's instructions.
  Injection was halted when substantial reflux occurred or when dangerous anastomosis filling was observed; after a two-minute stop to obtain a plug, injection could be started again if the embolic agent diffused towards the arterial prostate territory to obtain more effective penetration. Injection was stopped when intraprostatic vessels were occluded or if there was too much reflux around the catheter tip (Fig. 1). The microcatheter was then removed. A vascular closure device FemoSeal (Terumo, Tokyo, Japan) was routinely placed at the puncture site.
  Patients were monitored in the ambulatory surgery department for 6 h. They were discharged home with a prescription for 2 weeks of a prophylactic oral antibiotic and a nonsteroidal anti-inflammatory drug.
C) Noncontrast CT of the pelvis was performed within 4 weeks before PAE and within the month following embolization. NTE was defined as areas of high density visualized within the rectum, bladder, penis, or seminal vesicles on the CT scan performed 1 month after PAE.
  The images before and after embolization were reviewed independently by 2 board-certified radiologists (CR, FP) with 25 years and 2 years of experience, respectively. If there was a disagreement, the findings were discussed until a consensus was reached.
D) The safety of the prostatic embolization procedure with EVOH was assessed by collecting adverse effects. This collection was organized postoperatively by telephone contact with the patient on D2 and D6 postoperatively. At each visit (1 month-3 months), the subjects were questioned about the possible occurrence of side effects.
  Side effects were divided into early adverse effects (within 6 days after embolization) and late effects after this 6-day period. All the adverse effects linked to the embolization technique were collected: EVOH allows more distal embolization than the particles [1, 6], and one could expect a postembolization syndrome linked to more pronounced prostatic ischaemia, characterized by nausea, vomiting, fever, perineal pain, dysuria, haematuria, and urine retention. For this last symptom, it was also noted whether evacuating bladder catheterization was necessary within 12 h of the intervention.
  All side effects were classified according to the Clavien‒Dindo classification ((Mitropoulos D et 10.1016/j. s.d.) which divides postoperative complications into five grades from I to V, depending on the need for treatment: Grade I: any deviation from normal postoperative course, without any need for surgical, endoscopic, radiological or medical treatment, debridement of the wall abscess at the patient's bedside, or authorized treatment: antiemetics, antipyretics, analgesics, diuretics, electrolytes and physiotherapy; Grade II: need for pharmacological treatments other than those authorized above; indication for transfusion or total parenteral nutrition. Grade III: complication requiring surgical, endoscopic, or radiological treatment: grade III a: requiring treatment under local anaesthesia, grade III b: requiring treatment under general anaesthesia; Grade IV: threatening complications, including central neurological; ICU (Intensive Care Unit) indication: grade IV a: organ failure (including dialysis), grade IV b: multivisceral failure; Grade V: death.
E) Data were collected before PAE (baseline), after PAE, and at a scheduled follow-up visit 3 months after PAE. The evaluations included the International Prostate Symptom Score (IPSS) and quality of life (QoL).
F) Statistical Analysis: Continuous data are reported as the mean ± standard deviation, and categorical data are reported as absolute and relative frequencies. The evolution of IPSS and QoL scores was defined as the difference between the values at three months and baseline. The normality of these differences was tested using the Shapiro–Wilk test. In cases with a normal distribution, a paired Student’s t test was performed to compare the mean values; otherwise, the Wilcoxon rank-signed test was used. All tests were two-sided, and the significance level was set at 5%. Statistical analyses were performed using SPSS Statistics (version 11.0; SPSS, Chicago, Illinois, USA).

**Table 1:** Patient inclusion and exclusion criteria

Inclusion criteria	Exclusion criteria
More than 40 years of age	Biopsy-confirmed prostate cancer
LUTS attributed to BPH	Active urinary tract infection
LUTS duration ≥ 6 months,	Advanced atherosclerosis with tortuosity of the pelvic arteries
F**ailure to respond to standard pharmacotherapy**	Advanced renal failure^a^
IPSS > 18	Contrast hypersensitivity refractory to standard medications
IPSS-related QoL score > 2	
Prostate volume > 40 mL	

*LUTS* lower urinary tract symptoms, *BPH* benign prostatic hyperplasia, *IPSS* International Prostate Symptom Score, *QoL* quality of life

^a^Serum creatinine level of > 1.8 mg/dL or glomerular filtration rate of < 0 mL/min as estimated using serum creatinine levels, unless the patient was anuric and on dialysis

## Results

Between June 22 and January 2023, 10 consecutive patients were enrolled. Table 2 summarizes the demographics and baseline characteristics of the study population, and Table 3 displays the technical features of PAE with EVOH and short-term safety outcomes.

**Table 2:** Baseline characteristics of enrolled patients

Patients n	10
Age (Mean ± SD)	72.6 (63–82) SD 5.6
Urologic medical history, n (%)	
Yes	1 (1%)^a^
No	9 (9%)
Cardiovascular medical history, n (%)	6 (6%)
High blood pressure/diabetes	4 (4%)
Smoking	4 (4%)
Dyslipidaemia	4 (4%)
Anticoagulant ± Antiplatelet therapy, n (%)	6 (6%)
IPSS (Mean ± SD)	18.7 (8–32) SD 7.6
QoL (Mean ± SD)	5.3 (3–6) SD 1
Medication(s) for BPH n (%)^b^	10 (100%)
Prostate Volume, mL (Mean ± SD)	84 (50–170) SD 40
PSA, ng/mL (Mean ± SD)	3.94 (1–15) SD 3.9
Age (Mean ± SD)	72.6 ± 5.6
Urologic medical history, n (%)	
Yes	1 (1%)
No	9 (9%)
Cardiovascular medical history, n (%)	6 (6%)
High blood pressure/diabetes	4 (4%)
Smoking	4 (4%)
Dyslipidaemia	4 (4%)
Anticoagulant ± Antiplatelet therapy, n (%)	6 (6%)
IPSS Mean ± SD	18.7 ± 7.6
QoL Mean ± SD	5.3 ± 1
Medication for BPH n (%)	10 (100%)
Prostatic volume Mean ± SD	84 ± 40 mL
PSA Mean ± SD	3.94 ± 3.9 ng/mL

^a^Penis prosthesis

^b^Some patients received more than one drug, *IPSS* International Prostatic Symptoms Score, *QoL* quality of life, *BPH* benign prostate hyperplasia, *PSA* prostate-specific antigen

**Table 3:** Technical features and short-term safety outcomes

Variables	Data
General anaesthesia, *n* (%)	10 (100%)
Bilateral femoral approach, *n* (%)	10 (100%)
Type of embolization, *n* (%)	
Unilateral	1(10%)
Bilateral	9 (90%)
Total injected EVOH volume, mL	
Mean ± SD	0.8 (0.7–13) ± 0.4
Total PAE duration, min	
Mean ± SD	132 (120–150) ± 16
Fluoroscopic duration, min	
Mean ± SD	48 (32–55) ± 10
Radiation dose cgy.cm^2^	
Mean ± SD	52927 (26389–72,152) ± 10230
Complications according to the Clavien–Dindo score, *n* (%)	
IIIa	2 (20%)
NTE^a^, *n*	1
General anaesthesia, n (%)	10 (100%)
Bilateral femoral approach, n (%)	10 (100%)
Type of embolization, n (%)	
Unilateral	1 (10%)
Bilateral	9 (90%)
Total injected EVOH volume, Mean ± SD	0.8 ± 0.4 mL
Total PAE duration, Mean ± SD	132 ± 16 min
Fluoroscopic duration, Mean ± SD	48 ± 10 min
Radiation dose, Mean ± SD	52,927 ± 10,230 cGy.cm^2^
Complications according to the Clavien‒Dindo score, n (%)	
I	0 (0%)
II	0 (0%)
IIIa	2 (20%)
IIIb	0 (0%)
IVa	0 (0%)
IVb	0 (0%)
V	0 (0%)
NTE, n (%)	1 (10%)

^a^*NTE* nontarget embolization

Transfemoral access was performed in all patients. Bilateral PAE was technically successful in 9 patients, and unilateral injection was performed in one patient because of unilateral agenesis of the prostatic artery. No clogging of the microcatheters was observed. All patients were discharged on the same day.

**Fig. 2:**
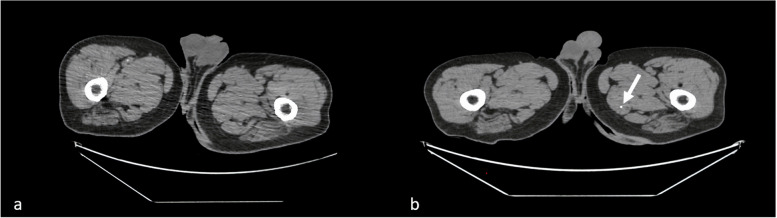
Noncontrast CT of the pelvis before (**a**) and after (**b**) prostatic embolization. Legend: Postembolization CT showing a punctiform NTE in the left pectineus muscle (white arrow)

The postoperative scanner showed a distribution of the embolization material in the two lobes of the prostate for all patients. One patient presented one punctiform and asymptomatic NTE in the pectineus muscle (Fig. 2).

None of the patients presented with pelvic pain and/or fever after embolization. Ninety percent of patients presented with urgency for 2 to 3 days. No patients had major adverse events. Two patients presented with postoperative urinary retention requiring bladder catheterization. Successful removal of the bladder catheter was accomplished in these two patients on D1 and D10.

After 3 months, the mean values for the IPSS and QoL score improved significantly (Table 4).

**Table 4:** PAE efficacy outcomes after 3 months

Variables	Baseline	3 months	Change (%)	*P* value
IPSS, Mean ± SD	18.7 ± 7.6	7.2 ± 4.3	-11.5 (61%)	< 0.01
QoL score, Mean ± SD	5.3 ± 1	1.9 ± 1.75	-3.4 (64%)	< 0.01

*IPSS* International Prostatic Symptoms Score, *QoL* quality of life

## Discussion

Some animal studies investigating arterial embolization with particles have reported the frequent presence of NTE [7]. In humans, Sakamoto et al. reported that more than 20% of complications of arterial embolization with particles were directly related to NTE [8]. In prostatic embolization procedures, NTE could be very common; thus, Brown et al. [3], using an embolic solution containing radiopaque microparticles and performing a postoperative CT scan, showed that NTE was observed in all treated patients. If all NTEs are not responsible for symptomatology [3], a correlation between sometimes serious side effects (penile ulceration, rectal or anal pain, haematuria, or rectal bleeding) observed after embolization and NTE can be obvious [9, 10]. NTE may be related to anatomic arterial variations and suboptimal catheter placement; it can also be secondary to the presence of dangerous anastomoses observed between the prostatic branches and surrounding arteries, such as the internal pudendal, rectal and vesical arteries [11]. Embolization with microparticles depends above all on vascular flow for target delivery, and in the absence of sufficient flow, such as in the setting of proximal vasospasm, reflux may occur with these embolic materials [12]. Several techniques have been described to avoid these NTEs: the PErFecTED technique [13] and occlusion of anastomoses with microcoils or gelatine sponges [14]. However, these dangerous anastomoses are not always easily detected during angiography, and the arterial anastomoses detected are variable depending on the injection rate, volume and pressure of the contrast agent used [11]. Similarly, the prior embolization of dangerous collaterals or the use of a protective balloon did not, according to Brown et al., prevent emboli outside the prostatic artery. The size of the particles also seems for some to influence the number of NTEs: the use of particles smaller than 300 μm would be associated with more complications [3, 15, 16]. In contrast, other authors [17, 18] have shown that the use of large particles increases the risk of arterial reflux. A randomized trial [15] comparing the use of 100–300 vs. 300–500-micron calibre particles did not show a significant difference between the two groups and concluded that the ideal particle size was yet to be determined [4]. Prostate artery embolization using N-butyl cyanoacrylate has been reported in three recent series [19–21]. In addition to clinical results comparable to those of prostatic embolization, the reported complications were minor and identical to those of embolization with particles. Ischaemic ulcerations of the penis (and 4 cases of transient erectile dysfunction) testifying to the existence of NTE were observed in two series, despite the radiopaque nature and greater ease of use of the liquid embolization material. The authors recognized that the results obtained could reflect the learning curve of the operators and the use of glue, which requires significant experience.

Prostatic embolization with EVOH was described by Chau et al. in 2018 in three patients [22]. In 2022, the feasibility and effectiveness of this technique was reported experimentally in dogs by Lucas Cav et al. [1], who compared particle embolization and embolization with ethylene vinyl alcohol copolymer (EVOH) and concluded that there was comparable efficacy in reducing the prostate volume and a possible benefit to EVOH embolization over particle embolization in terms of the risk of NTE. The rarity of NTEs after embolization with ethylene vinyl alcohol copolymer (EVOH) has been reported in cases involving hepatic embolization [23], head and neck embolization [24], and peripheral interventional radiology [25]. Prostatic embolization in our study showed a real efficacy of EVOH embolization comparable to the efficacy of the main published series and was associated with only one NTE. This result can be explained by the very good visualization of the product by fluoroscopy and by a better control of the gesture: the viscous nature of the product and its physical properties make it possible to stop the injection when it is not strictly intraprostatic and thus avoid NTE through dangerous anastomoses. Very comparable in terms of radiopacity with glue, the advantage of EVOH lies in the fact that it can be injected for a longer time, that it has greater cohesiveness and that the injection can be suspended for two minutes and restarted afterwards, allowing theoretically better arterial filling. EVOH is also said to be easier to use than glue, which requires experience and a longer learning curve [20, 23].

Moreira et al. [26] were one of the first to describe postembolization syndrome (PES) as the most common side effect of prostatic embolization, with a frequency of approximately 25% [27]. The two most frequent individual PES components were dysuria/urethral burning, local pain, and fever [27]. The symptoms vary in their severity and duration and can, if pronounced, be mistaken for urosepsis. Consequently, a subset of patients may need admission to the hospital for observation and symptomatic treatment with a combination of analgesics, antipyretics and antiemetics. EVOH embolization results in greater inflammation and more distal arterial occlusion than particle embolization [7, 28]. One could therefore expect a more intense PES in relation to parenchymal necrosis and more significant local inflammation. However, in our study, in addition to urgency in the first hours and 2 cases of acute urine retention, no other symptomatology was observed postoperatively: in particular, neither fever nor pelvic pain was present. In the literature, ischaemia and inflammation of the prostate after embolization are considered the cause of PES. The absence of pain and fever in our series, associated with the low number of NTEs, could raise questions about the aetiology of this syndrome, which may be correlated with extraprostatic ischaemic lesions linked to NTE. Confirmation of this result by other studies containing more patients is crucial to improve postoperative comfort and satisfaction.

Our study has limitations. First, it had a single-centre design. Second, there was a low number of patients. Third PAE with EVOH was not compared to another intervention or embolic agent. Fourth, the follow-up of 3 months was too short to draw conclusions on the long-term efficacy of the technique and the absence of later recanalization. Finally, we did not have uroflowmetric or postvoid residual volume data.

## Conclusions

PAE with EVOH is safe and associated with few NTEs. Further prospective studies with longer follow-ups are warranted to confirm the efficacy and safety of this embolic agent.

## Data Availability

The datasets used and/or analysed during the current study are available from the corresponding author upon reasonable request.

## Abbreviations

NTE: Nontarget embolization
PAE: Prostatic artery embolization
EVOH: Ethylene vinyl alcohol copolymer
BPH: Benign prostatic hyperplasia
LUTS: Lower urinary tract symptoms
PES: Postembolization syndrome
IPSS: International Prostate Symptom Score
QoL: Quality of life
